# Nanotechnology in Drug Delivery for Liver Fibrosis

**DOI:** 10.3389/fmolb.2021.804396

**Published:** 2022-01-11

**Authors:** Lihong Gu, Feng Zhang, Jinhui Wu, Yuzheng Zhuge

**Affiliations:** ^1^ Affiliated Drum Tower Hospital, Medical School of Nanjing University, Nanjing, China; ^2^ State Key Laboratory of Pharmaceutical Biotechnology, Chemistry and Biomedicine Innovation Center, Medical School of Nanjing University, Nanjing, China; ^3^ Jiangsu Key Laboratory for Nano Technology, Nanjing University, Nanjing, China

**Keywords:** chronic liver diseases, liver fibrosis, nanotechnology, nanomedicine, targeted drug delivery

## Abstract

Liver fibrosis is a reversible disease course caused by various liver injury etiologies, and it can lead to severe complications, such as liver cirrhosis, liver failure, and even liver cancer. Traditional pharmacotherapy has several limitations, such as inadequate therapeutic effect and side effects. Nanotechnology in drug delivery for liver fibrosis has exhibited great potential. Nanomedicine improves the internalization and penetration, which facilitates targeted drug delivery, combination therapy, and theranostics. Here, we focus on new targets and new mechanisms in liver fibrosis, as well as recent designs and development work of nanotechnology in delivery systems for liver fibrosis treatment.

## Introduction

**FIGURE 1:**
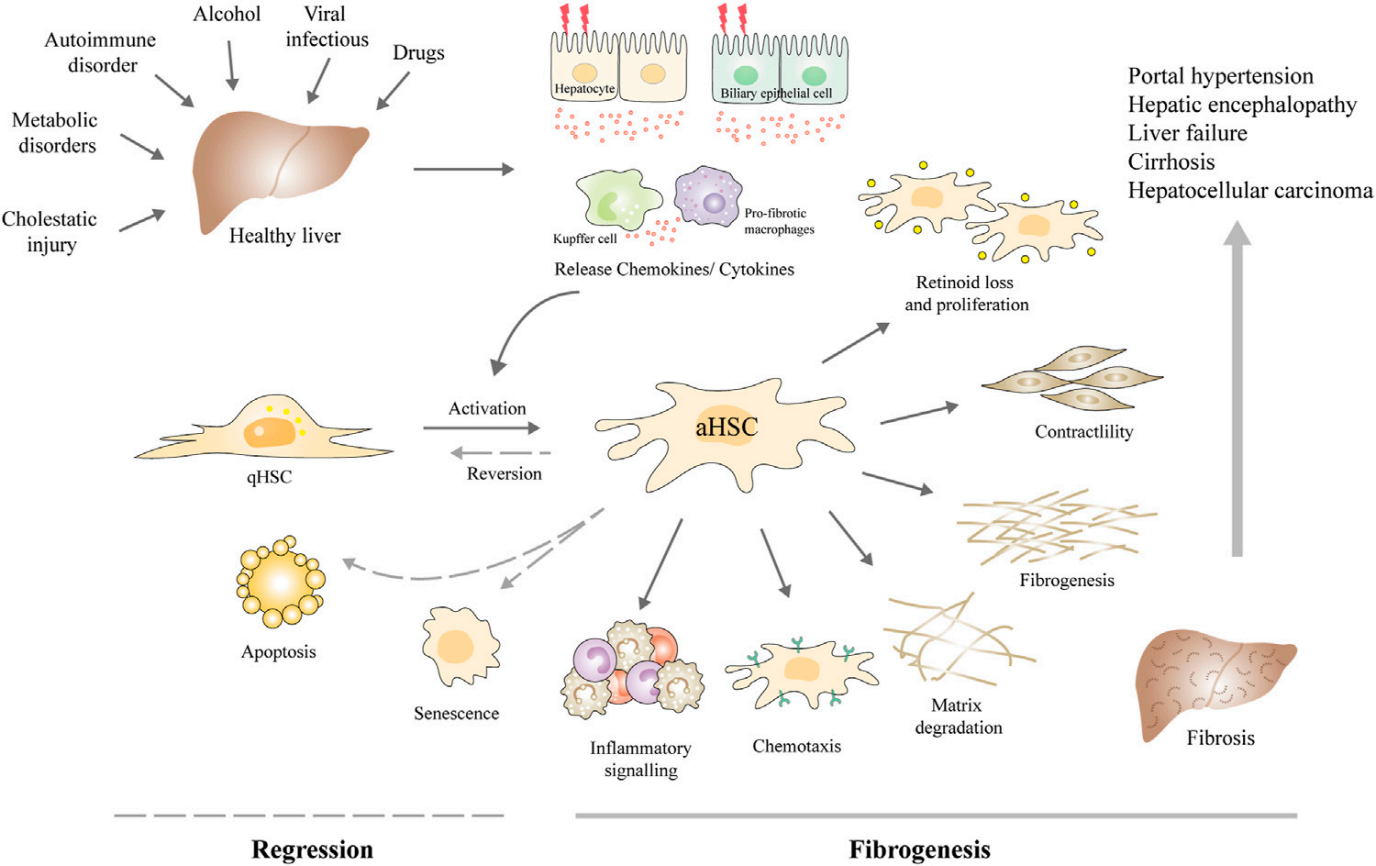
Schematic diagram of the central role of HSCs in the fibrogenesis and regression of liver fibrosis. Upon various chronic liver injuries, the hepatocytes and biliary epithelial cells can be damaged, together with Kupffer cells and macrophages, release chemokines, and cytokines to activate the HSCs. aHSCs are prone to present characteristics including retinoid loss, proliferation, contractility, ECM production, altered matrix degradation, chemotaxis, and expressing inflammatory signals. Liver fibrosis may develop into cirrhosis and even liver cancer, along with a suite of complications, such as portal hypertension, hepatic encephalopathy, and liver failure. Currently, the main antifibrotic therapy strategies are inhibiting HSC activation and proliferation, reversing the activation phenotype to the quiescent phenotype, or inducing HSC apoptosis and senescence. Abbreviations: qHSC, quiescent hepatic stellate cell; aHSCs, activated hepatic stellate cell.

Chronic liver diseases (CLD) remain a major concern for public health around the world. About 844 million people suffer and 2 million people die per year (Byass, 2014). Liver fibrosis is a common stage of various CLD, which is an important repair and pathological process caused by different etiologies, for instance, chronic viral infections (e.g., hepatitis B virus, hepatitis C virus), metabolic disorders, alcohol abuse, autoimmune insults, or cholestatic injury (Kisseleva and Brenner, 2021). In most cases, liver fibrosis is a kind of asymptomatic disease, which is usually clinically silent and progress slowly. With persistent damage and extracellular matrix (ECM) deposition, the liver is progressively hardening and stiffening over time followed by a progressive declined function. Then liver fibrosis may develop into cirrhosis and even liver cancer, along with a suite of complications, such as portal hypertension, liver failure, and hepatic encephalopathy (HE) (Parola and Pinzani, 2019) (Figure 1). The only effective therapy for terminal liver disease is liver transplantation.

Fortunately, many researches have revealed that liver fibrosis can be reversed in patients when the etiological factors are removed and in experimental rodent models when hepatic stellate cells (HSCs) are deactivated (Troeger et al., 2012; Lee et al., 2015). Therefore, early diagnosis and timely treatment of fibrosis is important for clinical management. Liver biopsy has been regarded as “gold standard” for the diagnosis and staging of liver fibrosis. However, it is invasive and has some significant complications, so patient acceptance is low (Bedossa and Carrat, 2009). Lacking accurate diagnostic techniques during long-term monitoring of progressing fibrosis and responses to therapy is a major challenge for optimizing disease treatment strategies (Bansal et al., 2016). Although, many research has discovered the etiology and pathogeny of liver fibrosis, and numerous therapeutic drugs are under development. Deactivating HSCs and expediting the clearance of myofibroblasts remain effective therapeutic strategies for the regression of liver fibrosis. Currently, the main antifibrotic therapy strategies are inhibiting the activation and proliferation of HSCs, reversing the activation phenotype to the quiescent phenotype, inhibiting HSCs autophagy, inducing cell apoptosis and senescence as well as immune clearance, or promoting ECM degradation (Ezhilarasan et al., 2018) (Figure 1). Pharmacotherapies like Chinese herbal medicines, chemical drugs, and monoclonal antibodies have been developed against these targets (Peres et al., 2000; de Oliveira et al., 2008; Ogawa et al., 2010). However, these traditional pharmacotherapies have inadequate therapeutic efficacy and unwanted side effects. For example, sorafenib and interferon-γ (IFNγ) have displayed great antifibrotic effects *in vitro* but present poor effects *in vivo*. No licensed pharmacotherapy is currently available for liver fibrosis. The development of therapeutic approaches with enhanced efficacy and targeted capabilities is in need. Determining the new mechanisms of liver fibrosis regression is also needed for identifying new therapeutic targets to treat liver fibrosis.

In recent decades, nanotechnology has greatly contributed to the design and apply of nanomedicine in terms of diagnosis and treatment for CLD. Well-designed nanostructures have specific targeting ability as well as diagnostic capability of liver fibrosis, which can be used as therapeutic agents, contrast enhancement agents, or nanoprobes for diagnosis (Li et al., 2018b). A lot of organic or inorganic nanoparticles (NPs) have been developed for liver fibrosis, including metal nanoparticles, lipid nanoparticles, polymer nanoparticles, and protein nanoparticles. The controllable size, shape, diverse components, and modifiable surface characteristics of nanoparticles bring superior advantages, including the prolonged circulation, improved internalization and penetration, controlled drug release, high contrast, improved drug pharmacokinetics, and reduced adverse reactions (Petros and DeSimone, 2010), which facilitate targeted drug delivery, combination therapy, and theranostics. For example, a study investigated a new theranostic nanomedicine, relaxin-PEGylated superparamagnetic iron-oxide nanoparticles, which can integrate diagnosis, and therapy in one platform (Nagorniewicz et al., 2019).

In this review, we focus on new targets and mechanisms, as well as recent designs and development work of nanotechnology in delivery systems for liver fibrosis treatment (Table 1). Major challenges and coping strategies in nanomedicine for liver fibrosis are also discussed.

**TABLE 1:** Preclinical nanomedicine for liver fibrosis.

Targeted structure	Nanoparticle formulation	Target cell, effects	Model	Reference
PDGFRβ	HMGB1-siRNA@SNALP-pPB	HSC, reduced proliferation, anti-inflammatory	TAA/CCl4-induced cirrhosis	Zhang et al. (2020)
	pPB-SSL-IFN-γ	HSC, inhibited proliferation, decreased fibrosis	TAA induced fibrosis	Li et al. (2012)
	GNR-AbPDGFRβ	HSC, decreased fibrosis, hepatic inflammation, and hepatocyte injury	CCl_4_-induced fibrosis	Ribera et al. (2021)
Sigma-1 receptor	AEAA-pRLN-LPD NPs	HSC, deactivated HSC, macrophage phenotype switch	CCl_4_-induced fibrosis, MCD- or CDAHFD-induced non-alcoholic steatohepatitis	Hu et al. (2021)
Integrin αvβ3	Vismodegib-cRGDyK-liposomes	HSC, inhibited hedgehog pathway signaling, reduced fibrosis	BDL/TAA induced fibrosis	Li et al. (2019)
	GMO-and miR-29b-loaded cRGD-PEG-PLGA NPs	HSC, cytotoxicity to aHSCs, inhibited production of collagen type Ⅰ	CCl_4_-induced fibrosis	Ji et al. (2020)
RBP	CGPVMs	HSC, inhibit collagen I accumulation	CCl_4_-induced fibrosis	Qiao et al. (2018)
	siCol1α1/siTIMP-1 VLNPs	HSC, promote collagen degradation, and inhibit collagen synthesis	CCl_4_-induced fibrosis	Qiao et al. (2020)
CD44	DOX-RA-CS micelles	HSC, downregulated collagen I production	CCl_4_-induced fibrosis	Luo et al. (2019)
	HA-UCNP@mSiO2@RBS	HSC, HSC apoptosis, liver fibrosis relief	CCl_4_-induced fibrosis	Liang et al. (2020)
Mannose receptor	TNF-α siRNA MTC NPs	Macrophage, reduced TNF-α production	LPS/d-GalN-induced hepatic injury	He et al. (2013)
PS	Cur-mNLCs	Macrophage, reduced fibrosis, increased HGF, and MMP2	CCl_4_ treated rat model	Wang et al. (2018)
ASGPR	ASGPR targeting tracer	Hepatocyte, quantify, and stage liver fibrosis	CCl_4_-induced fibrosis	Zhang et al. (2016)
	P-SPIONs	Hepatocyte, early diagnosis of liver fibrosis	CCl_4_-induced fibrosis	Saraswathy et al. (2021)
Passive target	S1PR_2_-siRNA GeRP	Macrophages, decreased NLRP3 inflammasome activation, attenuated hepatic inflammation, and fibrosis	BDL/MCDHF/CCl_4_-induced fibrosis	Hou et al. (2020)
	TSG-6@CaP@BSA NPs	Macrophage, M2 polarization, increased MMP12 expression	CCl_4_-induced fibrosis	Wang et al. (2020)
	PD-MC	Multiple cell types, reduced hepatocyte apoptosis, averted activation of macrophages, and HSCs	CCl_4_-induced fibrosis	Lin et al. (2020)

Abbreviations: PDGFRβ, platelet-derived growth factor receptor β; HMGB1, high mobility group box-1; SNALP, stable nucleic acid lipid nanoparticles; pPB, “C*SRNLIDC*” (peptide *vs.* PDGFβR); HSC, hepatic stellate cell; TAA, thioacetamide; CCl4, carbon tetrachloride; SSLs, sterically stable liposomes; IFN-γ, interferon-γ; GNR-AbPDGFRβ, PDGFRβ-antibody-conjugated gold nanorods; AEAA, aminoethyl anisamide; pRLN, plasmid DNA (pDNA) that encoded RLN; LPD, lipid–protamine–DNA; MCD, methionine-choline-deficient; CDAHFD, choline-deficient, l-amino acid-defined high-fat diet; cRGDyK, Cyclo [Arg-Gly-Asp-DTyr-Lys]; BDL, bile duct ligation; GMO, germacrone; PEG-PLGA, poly(ethylene glycol)-block-poly(lactide-co-glycolide); RBP, retinol binding protein; CGPVMs, silybin/siCol1α1 co-loaded core-shell polymer micelles; VLNPs, vitamin A-decorated and hyperbranched lipoid-based lipid nanoparticles; DOX, doxorubicin; RA, retinoic acid; CS, chondroitin sulfate; HA, hyaluronic acid; UCNP, upconversion nanoparticle; mSiO2, mesoporous silica; RBS, Roussin’s black salt; MTC, Mannose-modified trimethyl chitosan-cysteine; LPS, lipopolysaccharide; d-GalN, d-galactosamine; PS, phosphatidylserine; Cur, curcumin; mNLCs, PS-modified nanostructured lipid carriers; HGF, hepatocyte growth factors; ASGPR, asialoglycoprotein receptor; P-SPIONs, pullulan stabilized iron oxide nanoparticles; S1PR2, sphingosine 1-phosphate receptor 2; GeRP, glucan–encapsulated siRNA particle; NLRP3, NOD-, LRR-, and pyrin domain-containing protein 3; TSG-6, tumor necrosis factor stimulated gene 6; CaP, calcium phosphate; BSA, bovine serum albumin; MMP12, matrix metalloproteinase 12; PD‐MC, polydatin‐loaded micelle.

## Cell Targets in Liver Fibrosis

**FIGURE 2:**
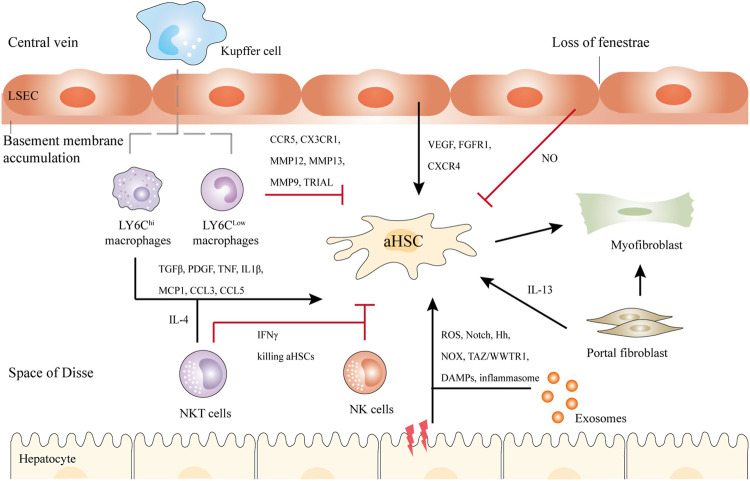
Schematic illustration of the crosstalk between HSCs and Kupffer cells, NK cells, NKT cells, hepatocytes, bone marrow-derived macrophages, and LSECs in liver fibrosis. Briefly, upon liver injury, hepatocytes undergo damage, and inflammatory, releasing DAMPs, exosomes, etc. LY6C^hi^ macrophages activate HSCs by TGFβ, IL1β, and other cytokines, while LY6C^low^ macrophages show antifibrotic ability *via* CX3CR1, MMP12, MMP13, etc. NKT cells and NK cells secret IFNγ to kill aHSCs. NKT cells can also activate HSCs through IL-4. In addition, LSECs undergo capillarization and accumulation of basement membranes. They can promote either liver regeneration or fibrosis. Another population of myofibroblasts is the portal fibroblasts, which can also activate HSCs in cholestatic liver. Black and red arrows represent promotion or inhibition of aHSCs respectively.

Various key molecular mechanisms resulting in liver fibrosis have been revealed, such as chronic hepatocyte injury, endothelial barrier damage, inflammatory cytokines release, recruitment of bone marrow-derived cells (BMDCs), secretion of transforming growth factor-β (TGFβ) by macrophages, HSC activation, excessive accumulation of ECM, as well as the production of fibrous scars (Bataller and Brenner, 2005). Fiber regression is the key to stopping fibrosis progression. Upon the removal of the above factors, the fibrosis will regress, along with the decreased pro-inflammatory or profibrogenic cytokines, disappeared aHSCs, increased collagenolytic activity, suppressed ECM production, and reduced fibrous scars (Kisseleva and Brenner, 2021). The crosstalk between hepatic myofibroblasts, macrophages, injured hepatocytes, and other cell types make contributions to these mechanisms (Figure 2).

### Hepatic Stellate Cells

The activation of HSCs is regarded as the central link in liver fibrosis and therefore represents a major target for antifibrotic therapy (Higashi et al., 2017). Quiescent HSCs are located at the Disse space, an area between hepatocytes and sinusoidal endothelial cells (LSEC), with the proportion of 5–8% among the hepatocytes. Under normal physiological conditions, HSCs are in a quiescent state and responsible for storing retinoid lipid droplets (mainly vitamin A) in liver (Senoo et al., 2017). Upon liver injury, accompanied by hepatocyte inflammation, macrophages will recruit and transform the resident quiescent HSCs to a highly activated, proliferative, and contractile myofibroblast-like phenotype (Mederacke et al., 2013; Tacke and Zimmermann, 2014) (Figure 1). aHSCs are prone to lose lipid droplets, upregulate α-smooth muscle actin expression, and migrate to the injury site to secrete ECM to repair the damaged liver. As time goes on, the excessive accumulation of ECM and matrix metalloproteinases (MMPs) will induce remodeling of liver tissue, and the fibrous scars will eventually replace the normal tissue.

Actually, a panoply of signals drive HSCs activation. The key proliferative and fibrogenic pathways leading to fibrosis consist of TGFβ, vascular endothelial growth factor (VEGF), platelet-derived growth factor (PDGF), and connective tissue growth factor (CTGF) (Tsuchida and Friedman, 2017). It is generally accepted that TGFβ is the most potent profibrogenic cytokine in the activation process of HSCs (Hellerbrand et al., 1999). TGFβ drives the activation of HSCs mainly *via* SMAD2-SMAD3 signaling to promote transcription of type Ⅰ and Ⅲ collagen (Dewidar et al., 2019). Genetically overexpressing TGFβ spontaneously induce liver fibrosis, whereas genetic deletion or pharmacological blockade of TGFβ could ameliorate fibrosis in mice (Hellerbrand et al., 1999; de Gouville et al., 2005). However, persistent depletion of TGFβ has adverse effects like poor wound healing and tumor genesis. Hedgehog signaling (Omenetti et al., 2011), innate immune signaling (especially Toll-like receptors and cytokines) (Seki et al., 2007), G protein-coupled receptors (Granzow et al., 2014), reactive oxidative stress (ROS) (Novo et al., 2011) have been implicated in HSC activation. A study using clinical liver biopsies and animal models revealed a new profibrotic function of interleukin (IL)-17A and IL-22 in a TGFβ-depend manner (Fabre et al., 2018). Blocking either IL-17 or IL-22 by antagonists resulted in reduced fibrosis. Autophagy provides HSCs with energy substrates and the process is related to upreguated endoplasmic reticulum stress (Hernández-Gea et al., 2013). Epigenetic signals regulate both activation and deactivation of HSCs. Nuclear receptors like peroxisome proliferator-activated receptor gamma (PPARγ), farnesoid X receptor (FXR), and liver X receptor (LXR) modulate the inactivation of HSC (Tsuchida and Friedman, 2017).

### Portal Fibroblasts

Many cell populations, for example, HSCs, bone marrow-derived fibroblasts, portal fibroblasts, and mesenchymal cells contribute to ECM accumulation, the primary sources of the activated myofibroblasts that secrete ECM proteins are HSCs and portal fibroblasts (Kisseleva, 2017). It depends on the etiology of liver fibrosis, the major sources of myofibroblasts can differ (Iwaisako et al., 2014). aHSCs contribute >87% of myofibroblasts in hepatotoxic (carbon tetrachloride, CCl_4_) liver, while activated portal fibroblasts (aPFs) are the major source in cholestatic liver (bile duct ligation, BDL), contributing >70% of myofibroblasts upon injury. Mesothelin/mucin 16 signaling might play a vital role in this biology (Koyama et al., 2017). With progressive injury by BDL, the role of aPFs lessens, HSCs gradually gain the upper hand and contribute to the myofibroblasts. Upon cholestatic injury, taurocholic acid directly stimulates COL1A1 expression in aPFs. Furthermore, IL-25 can stimulate the secretion of IL-13 in aPFs, which provides stimulus signals to HSCs (Liu et al., 2011). This understanding suggests that targeting aPFs might provide new directions for the regression of liver fibrosis.

### Macrophages

The activation of macrophages also plays a key role in the fibrogenesis process. Macrophages in liver can be divided into resident Kupffer cells (KCs) and monocyte-derived macrophages. KCs have phagocytic ability and anti-inflammatory functions (Krenkel and Tacke, 2017). It is also reported that KCs have a profibrogenic role. Firstly, KCs recruit proinflammatory and profibrogenic macrophages through CCL2 secretion in the early stage of liver injury. Secondly, KCs secret TGFβ and PDGF to directly activate HSCs. Thirdly, KCs establish a profibrogenic niche by producing proinflammatory cytokines and chemokines, for instance, IL-1β, tumor necrosis factor α (TNFα), IL-6, and CCL5, which may interact with HSCs (Wen et al., 2021).

After injury, in response to inflammatory signals, BMDCs migrate to inflamed site, and differentiate into macrophages, which play a dual role in the fibrogenesis and regression process. During fibrogenesis, macrophages display a LY6C^hi^ phenotype and produce cytokines and chemokines to activate HSCs, including TGFβ, PDGF, TNF, IL-1β, monocyte chemoattractant protein-1 (MCP1), CCL3, and CCL5 (Wen et al., 2021). The recruitment of this type of macrophages is CCR2-dependent in CCl_4_ treated mice. CCR2-deficient mice presented an impaired recruitment of LY6C^hi^ macrophages, reduction of aHSCs, and diminished fibrosis (Karlmark et al., 2009). During fibrosis regression, macrophages display a LY6C^low^ phenotype expressing high levels of CCR5 and CX3CR1 and produce MMPs (MMP12 and MMP13) to promote fibrosis degradation (Krenkel and Tacke, 2017). CX3CR1 binds with its ligand to inhibit the inflammatory property of macrophages. Furthermore, LY6C^low^ macrophages promote HSCs apoptosis *via* MMP9 and TNF-related apoptosis-inducing ligand (TRAIL) (Taimr et al., 2003). Moreover, the decreased tissue inhibitor of metalloproteinase (TIMP) expression contributes to the increased activity of collagenolytic enzymes. TIMPs help aHSCs survive *via* the downregulation of pro-apoptotic BAX and PUMA and upregulation of BCL2 (Parsons et al., 2004). The depletion of macrophages led to a failure of ECM degradation in CCl_4_-treated mice (Duffield et al., 2005), suggesting that a more delicate and circumspect therapeutic approach is in need to effectively target different subpopulations of macrophages for treating liver fibrosis.

Recently, the critical role of c-Rel (the subunit of NF-κ-B) in regulating metabolic changes required for inflammatory and fibrogenic activities of macrophages and hepatocytes was reported by Leslie et al. (2020). In CCl_4_-induced liver fibrosis, independent knockout of Rel in macrophages or hepatocytes inhibited liver fibrosis, while combined knockout in both cell types had an additive antifibrosis effect. They also identified Pfkfb3 as a key downstream mediator in this process. Targeting the c-Rel-Pfkfb3 axis might be a multiple cell targeting strategy for antifibrotic treatment.

It is worth mentioning that exosomes also play a role that cannot be ignored in the regulation by macrophages in fibrosis. Macrophages and neutrophil can release exosomes to alter liver functions. For instance, miR-233 can be delivered by macrophages *via* exosomes to many cells, including hepatocytes, controlling inflammation in liver diseases (Ismail et al., 2013; Ye et al., 2018). A recent work by Hou et al. (2021) discovered that IL-6 signaling played a critical role in controlling liver fibrosis in a macrophage-specific way. IL-6 promotes macrophages-derived miR-223-enriched exosomes to suppress the several miR-223-targeted genes expressed in hepatocytes in a nonalcoholic steatohepatitis (NASH) model.

### Other Immune Cells

Besides macrophages, numerous other immune cell populations infiltrate in the fibrotic liver. Natural killer (NK) cells display an antifibrotic property generally by killing aHSCs through IFNγ and inducing HSC apoptosis in a TRAIL- and NKG2D-dependent manner (Jeong et al., 2011; Glässner et al., 2012). In murine models, enhancing NK cell activity *via* inducing the expression of IFNγ promotes fibrosis resolution (Krizhanovsky et al., 2008). By the way, NK cells also facilitate fibrosis resolution by clearing senescent myofibroblasts.

Natural killer T (NKT) cells also play a dual role in liver fibrosis. On the one hand, same as NK cells, NKT cells show antifibrotic functions by directly killing aHSCs through IFN-γ secretion (Park et al., 2009). On the other hand, in response to liver injury, CXCR6^+^ NKT cells promote fibrogenesis in liver. In fibrotic livers of Cxcr6 (-/-) mice, the infiltration of macrophage and expression of inflammatory cytokines (e.g., IFN-γ, TNF-α, and IL-4) were reduced, suggesting that hepatic NKT cells maintain hepatic inflammation and fibrogenesis by providing essential cytokines (Wehr et al., 2013).

The neutrophil extracellular traps (NET) also play a certain role in CLD. Upon liver injury, neutrophils migrate into the injury site, dismantle the damaged vessels, and create channels for vessel growth. Concerning nonalcoholic fatty liver disease (NAFLD), both Treg cells and Th22 cells seem to present an overall tempering effect, while Th17 cells induce more liver damage and promote fibrosis. Th1 cells secrete IFN-γ to reduce liver fibrosis, while Th2 cells can promote fibrosis by activating HSCs *via* cytokine release (Wynn, 2004). Little is known about the role of innate T cells in liver fibrosis and further investigations are required (Bartneck, 2021).

### Hepatocytes

Upon liver injury, hepatocytes generate inflammation and fibrosis. An increasing number of mediators, including ROS, Notch (Zhu et al., 2018), Hh ligands (Chung et al., 2016), NADPH oxidase (NOX) (Lan et al., 2015), and TAZ/WWTR1 (Wang et al., 2016), facilitate this process. Furthermore, exosomes containing micro RNAs secreted by injury hepatocytes might activate HSCs (Lee et al., 2017). Hepatocyte-derived exosomes promote fibrinolysis by suppressing the activation of HSCs, inhibiting macrophage activation and cytokine secretion, and inducing ECM degradation, and remodeling (Chen et al., 2019). Damage-associated molecular patterns (DAMPs) released from hepatocytes and inflammasome consisting of NLRP3, NACHT, LRR might promote HSC activation and liver fibrosis directly or indirectly (Wree et al., 2014). It is suggested that inflammation is a necessary condition for fibrosis.

### Liver Sinusoidal Endothelial Cells

Liver sinusoidal endothelial cells (LSECs) are fully differentiated in normal liver and have the capacity to maintain HSC quiescence via paracrine factors like nitric oxide (NO) (Deleve et al., 2008). In fibrotic livers, LSECs undergo capillarization and dedifferentiation, characterized by loss of fenestrae and basement membrane accumulation, become vasoconstrictive, proinflammatory and prothrombotic, and lose their ability of suppressing HSC activation. Additionally, fenestrae loss and basement membranes development prevent hepatocytes oxygenation, causing apoptosis and necrosis, which ultimately induce DAMP secretion to activate HSCs (Gracia-Sancho et al., 2021). Reduction of NO bioavailability and endothelial NO synthase (eNOS) activity, together with increased ROS-mediated scavenging of NO, all lead to HSC activation, and ECM deposition in CLD (Gracia-Sancho et al., 2021). VEGF-stimulated NO can activate soluble guanylate cyclase (sGC). The activator of sGC can restore capillarization and return HSCs to the quiescent state in rats (Xie et al., 2012). Depending on the LSEC response to liver injury, LSECs can promote liver regeneration by the CXCR7-ID1 pathway, or promote liver fibrosis *via* fibroblast growth factor receptor 1 (FGFR1)-CXCR4 pathway (Ding et al., 2014).

## Challenges in Pharmacotherapy for Liver Fibrosis

Even though so many key targets related to liver fibrosis have been discovered, there is no available single therapeutic agent to date. Various innovative antifibrotic attempts such as IFNγ, interleukin 10, and angiotension Ⅱ antagonists have shown promising results in preclinical trials, but failed in clinical trials (Bartneck et al., 2014). One major reason is likely that traditional formulations lack the specific delivery of the respective molecules. For example, IFNγ has valid antifibrotic activities, but has proinflammatory effects on macrophages (Mosser and Edwards, 2008). Since liver fibrosis is a complex process involving multiple cell types (HSCs, macrophages, LSECs, and NK cells), it is useless to target one cell type with a single pharmacological agent. This is also a reason for several antifibrotic drugs like simtuzumab who showed promising effect in preclinical experiments but failed in clinical translation. Moreover, biological differences exist between animal models and patients. So far, the targeted antifibrotic drugs neglect the pleiotropic potential of myofibroblasts (for example, activation, proliferation, releasing profibrogenic factors, and collagens) and only aim at a single mode of actions, such as, as PPARγ agonists, TGFβ inhibitors, PDGF inhibitors, etc. (Devaraj and Rajeshkumar, 2020). Many compounds derived from herbal and synthetic compounds such as silymarin and curcumin have shown promising effects for liver fibrosis. However, their poor bioavailability and water solubility, lack of specific targeting, and extra hepatic toxicity limit their application (Ezhilarasan et al., 2014).

Nanomedicines provide novel therapeutic opportunities for drug delivery in antifibrotic therapies. Nanotechnology combines the characteristics attributed to monoclonal antibodies and small molecule drug. Nanomedicine consists of the active component, for instance, small interfering RNA (siRNA), microRNA (miRNA), small molecule drug, cytokine, or protein, containing the delivery vehicle, such as liposome, protein carrier, polymeric nanoparticles, or micelles, which is guided to the specific site of the body by attaching of targeting ligands, based on ligand-receptor interactions. They may overcome many hurdles that traditional pharmacotherapy could not conquer. For instance, the drug concentration in the target cell or tissue is upregulated due to the specific targeting, while reduces adverse effects on other cell types. Additionally, nanomedicines can overcome biological barriers due to their controllable size and shape, protect the drug from being metabolized and prolong drug circulation in the bloodstream, and alter pharmacological features of the delivered drug (Bartneck et al., 2014). In preclinical models, numerous nanoformulations were explored for the treatment of liver fibrosis.

Current antifibrotic nanodrug therapies mainly focus on targeting HSCs, macrophages, NK cells, and LSECs, etc. Because the crosstalk between these cell populations is regarded as major contributor to liver fibrosis. The current cell therapy strategies are as follows: 1) suppressing the activation or proliferation of HSCs, promoting the apoptosis or senescence of aHSCs, switching from activated to quiescent state, inhibiting the secretion of ECM protein or promoting the expression of MMPs; 2) suppressing the production of pro-inflammatory and profibrotic factors by immune cells, or promoting the switch from fibrogenic to collagenolytic phenotype in macrophages; 3) protecting hepatocytes from injury or enhancing hepatocyte regeneration; 4) preventing the capillarization of LSECs. The following section deals with nanoparticles-based drug delivery strategies for liver fibrosis.

## Nanodrug Delivery System Targeting Hepatic Stellate Cells

In the last few years, numerous drug delivery approaches for specific-targeting of HSCs have been studied, for example, liposomes, protein-based delivery systems, viral vectors, and inorganic delivery systems, which can reduce drug adverse effects and further improve therapeutic effects of drugs.

### Targeting Platelet-Derived Growth Factor Receptor

**FIGURE 3:**
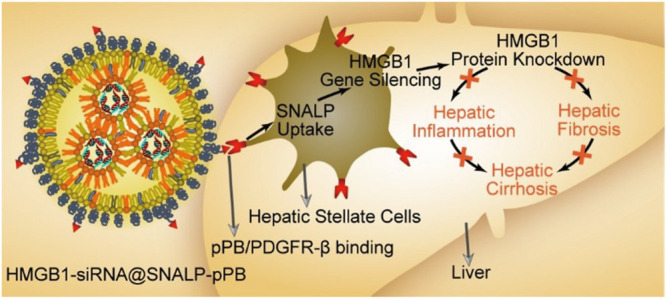
Schematic diagram of the HMGB1-siRNA@SNALP-pPB nanoparticle targeting HSCs to silence HMGB1 to show antifibrotic and anti-inflammatory effects for hepatic cirrhosis (Zhang et al., 2020).

PDGF receptor (PDGFR) is a dimer containing two related chains connected by disulfide bonds, which is specifically overexpressed on aHSCs. In view of this, Beljaars et al. developed a receptor-recognizing cyclic peptide-modified albumin (pPB) peptide “C^∗^SRNLIDC^∗^” for targeting HSCs (Beljaars et al., 2003). Li et al. (2012) took use of the pPB peptide-modified sterically stable liposomes encapsulating IFNγ (pPB-SSL-IFN-γ) to specifically target HSCs. The NPs suppressed the proliferation of HSCs *in vitro* and showed decreased fibrosis in thioacetamide treated mice. The drug delievery system prolonged circulation half-life of IFNγ and decreased its side effects in fibrotic livers. Recently, Zhang et al. (2020) constructed pPB peptide-modified stable nucleic acid lipid NPs loading HMGB1 (High mobility group box-1)-siRNA (HMGB1-siRNA@SNALP-pPB) to effectively treat liver cirrhosis by their dual antifibrotic and anti-inflammatory abilities (Figure 3). HMGB1 protein is known as a fibroblast chemokine and pro-inflammatory factor, which promotes the proliferation of HSCs and facilitates hepatic inflammation and fibrosis. The targeted nanoparticles with both antifibrotic and anti-inflammatory effects prolonged the survival of cirrhotic mice significantly, suggesting that combination of multiple therapeutic targets would be more effective.

In addition to peptide, specific targeting mediated by antibodies has been applied for HSCs too. Inorganic delivery systems like Gold NPs can be developed into different shapes like nanocages, nanorods, and nanosatellites due to the surface characteristics and controlled particle size. Recently, Ribera et al. developed gold nanorods with surface modified anti-PDGFRβ to specifically target aHSCs (Ribera et al., 2021). Meanwhile, the gold nanorods can induce localized near infrared light (NIR)-mediated thermal ablation due to the photothermal properties, along with aHSC targeting, hepatic inflammation reduction, and fibrosis regression were observed in the CCl_4_-induced fibrosis in mice. Moreover, inorganic nanomaterials themselves such as titanium dioxide (TiO_2_) NPs and silicon dioxide (SiO_2_) NPs demonstrate antifibrotic properties *in vitro* through acting on HSCs (Peng et al., 2018).

### Targeting Sigma-1 Receptor

Relaxin (RLN) used to be considered as an antifibrotic peptide hormone which can directly reverse HSC activation for fibrosis regression. Its primary receptor, relaxin receptor family peptide-1 (RXFP1), is upregulated on aHSCs. The binding of RLN and RXFP1 can initiate the NO signalling against profibrogenic pathways (Fallowfield et al., 2014). Hu et al., 2019 engineered RLN-plasmid (pRLN)-loaded lipid-calcium-phosphate NPs (LCPs), surface modified with AEAA (aminoethyl anisamide, ligand for the sigma-1 receptor, which is highly expressed on aHSCs), which can locally secret RLN in liver. The *in situ* enforced RLN expression by transfected aHSCs transformed themselves to quiescent phenotype and reduced ECM accumulation. In another study (Hu et al., 2021), the authors found relaxin gene therapy could reduce liver fibrosis *in vivo*, but the transmit of quiescence of aHSCs failed *in vitro* experiment. Moreover, relaxin is expressed by hepatic macrophages and on its binding, macrophages change from the pro-fibrosis to the pro-resolution phenotype via cAMP–PKA–CREB (cAMP, cyclic adenosine monophosphate; PKA, protein kinase A; CREB, cAMP-responsive element binding protein) pathway by activating Nur77 in macrophages. In view of this, they developed lipid–protamine–hyaluronic acid (LPH) nanoparticles encapsulate the relaxin gene and miR-30a-5p mimic, with surface modified AEAA, which show synergistic antifibrotic effects in rodent models. Here, miR-30a-5p derived from exosomes secreted by relaxin-educated macrophages can deactivate HSCs. The study provided a combinatory gene therapy involving macrophage phenotype switch, took into consideration the crosstalk between pro-resolution macrophages and aHSCs, synergistically and safely regressed liver fibrosis.

### Targeting Integrin αvβ3

Integrins are receptors for most adhesion proteins like fibronectin and collagen type Ⅵ, take responsibility for the interaction between ECM and cells. They all contain the arginine-glycine-aspartic sequence (RGD) peptide. The RGD peptide -modified nanodelivery systems have been widely applied for HSC targeting.

Recent work by Li et al. (2019) prepared liposomes containing the cyclic peptides [cRGDyK, Cyclo (Arg-Gly-Asp-DTyr-Lys)] with high affinity to αvβ3 to specifically target aHSCs, but not quiescent HSCs, which overcomed the lack of specificity in the previous RGD-modified systems that target both quiescent and activated HSCs. Vismodegib (VIS), a Hh inhibitor, has been reported to attenuate hepatic fibrosis by inhibiting HSC activation (Philips et al., 2011). The cRGDyK modified SSLs raised therapeutic efficacy of VIS by alleviating undesirable properties, such as water insolubility, short half-life, and off-target effects. Ji et al. (2020) took use of this cyclic peptides to modify germacrone (GMO)-and-miR-29b-loaded nanoparticles, which exhibited great cytotoxicity to aHSCs and suppressed production of collagen Ⅰ. In addition, cRGDyK used to be employed as an imaging modality for liver fibrosis (Li et al., 2016). It is a useful MRI tracer and can assess the extent of liver fibrosis non-invasively and quantitatively. It seems that cRGDyK-modified NPs act as an effective platform for both treatment and diagnosis of liver fibrosis.

### Targeting Retinol-Binding Proteins

HSCs contain most vitamin A (VA) of the body, accounting for 80%, and it can be selectively transported into HSCs by retinol binding protein receptor (RBPR) and cell retinoic acid binding protein (CRABP) overexpressed on the surface of HSCs (Lee and Jeong, 2012). Therefore, VA-based delivery systems have been developed for liver fibrosis.

Qiao et al. (2018) developed HSC-targeted NPs grafting VA for co-delivery of chemical (silibinin) and genetic (siCol1α1) drugs, which inhibit collagen I accumulation synergistically in fibrogenesis. The team followed a tradition of multi-target therapy and prepared a lipid delivery system which carried dual siRNAs intended to both promote collagen degradation (by siTIMP-1) and inhibit collagen synthesis (by siCol1α1) (Qiao et al., 2020). They use helper lipoids (Chol-PEG-VA) for HSCs targeting and amphiphilic cationic hyperbranched lipoids (C15-PA) for siRNA complexation to generate vitamin A-decorated and hyperbranched lipoid-based lipid nanoparticles (VLNPs). CT-VLNPs showed reduced collagen accumulation in treated mice to almost that seen in normal one. The combined therapy of co-delivering multi-target drugs achieved a substantial ideal effect, and providing a new direction for the treatment of liver fibrosis in future.

### Targeting CD44

CD44 used to be regarded as a cell surface adhesion receptor highly expressed in many cancers. Later, it was discovered the high expression on aHSCs and it can interact with chondroitin sulfate (CS) and hyaluronic acid (HA) (Fujimoto et al., 2001). Therefore, CS has been suggested as a suitable candidate material to fabricate delivery systems for HSC-targeting. Recently, Luo et al. (2019) developed DOX-RA-CS (DOX, doxorubicin; RA, retinoic acid; CS, chondroitin sulfate) micelles co-delivery system for the treatment of CLD. The micelles preferentially deposited in the Golgi apparatus, destroyed the Golgi structure, and ultimately reduced collagen I production. The micelles exhibited synergistic antifibrotic effects in the CCl_4_-treated rat model. In another study by Liang et al. (2020), HA was used as a candidate for HSC-specific drug delivery. Upconversion nanoparticle (UCNP) cores modified with HA and Roussin’s black salt (RBS) were enveloped in mesoporous silica shells (HA-UCNP@mSiO2@RBS), which can target HSCs and locally release NO when exposing under near NIR. The release of NO can trigger HSC apoptosis and fibrosis regression.

In addition, it has been reported that CD44 is a key player in non-alcoholic steatohepatitis (NASH) (Patouraux et al., 2017). It can regulate macrophage polarization and infiltration in liver to enhance the progression of NASH. Targeting CD44 might be a potential therapeutic strategy for NASH and related fibrosis.

## Nanodrug Delivery System Targeting Immune Cells

After systemic administration, NPs enter the blood stream, interact with serum proteins non-specifically, and resident macrophages of the endothelial network will remove NPs larger than 200 nm and negatively charged. Kupffer cells and LSECs in the space of Disse are inherent components of the reticuloendothelial system (RES). The diameter of the sinusoidal endothelial fenestrum is approximately 100–200 nm (Xing et al., 2021). The previous study reported that the NPs encapsulated bovine serum albumin (BSA) ranged 100–200 nm had good liver targeting ability. Nanomedicines targeting the immunologically relevant RES have been well summarized in another review by Matthias Bartneck (Bartneck, 2021).

Many nano-delivery systems have been developed for targeting macrophages, such as solid-lipid, liposomes, and polymeric NPs (Colino et al., 2020). NPs deliver drugs to macrophages by passive and active targeting. Lipoplex-based transfection has been applied for macrophage targeting, glucan encapsulating sphingosine 1-phosphate receptor 2 (S1PR2)-siRNA NPs could attenuate hepatic inflammation and fibrosis by reducing the activation of NLRP3 inflammasome (Hou et al., 2020). Transplantation of mesenchymal stem cells (MSCs) has been regarded as a promising antifibrotic strategy but facing some clinical controversies. Wang et al. (2020) found tumor necrosis factor stimulated gene 6 (TSG-6) was a major antifibrotic cytokine in MSCs. In view of the easily capture of NPs by the RES of the liver, they prepared TSG-6@CaP@BSA (CaP, calcium phosphate) NPs for antifibrotic treatment. The NPs induced M2 polarization of macrophages and increased the expression of MMP12, which could inhibit HSC activation and suppress release of pro-inflammatory cytokines.

The previous work by He et al. (2013) developed trimethyl chitosan-cysteine (MTC) conjugated anti-TNF-siRNA NPs modified by mannose, which are mostly taken by macrophages. In addition, macrophages can precisely identify phosphatidylserine (PS), an anionic phospholipid expressed by apoptotic cells. Wang et al. (2018) constructed PS-modified nanostructured lipid carriers (mNLCs) containing curcumin (Cur-mNLCs) for liver fibrosis in a CCl_4_ treated rat model. The PS-modification enhanced the uptake of NPs by macrophages in diseased liver, leading to fibrosis regression and upregulation of MMP2 and hepatocyte growth factors (HGF).

Therefore, the unspecific uptake of nanoparticles by immune cells like macrophages makes these cells an interesting target cell for nanomaterials. The immune cells act as a double-edged sword for drug delivery in liver fibrosis, where macrophages are easily targeted, but there is a risk of being cleared before they reach other cell types. Hence, more powerful and more unique targeting for specific subpopulations need further investigation.

## Nanodrug Delivery System Targeting Hepatocytes

In fibrotic liver, hepatocytes damage may have serious effect on liver function, such as, lipid, protein, sugar metabolism, and liver detoxification. Nanodrug delivery systems usually carry liver-protecting drugs to hepatocytes for maintaining the function of liver. Galactose-modified delivery systems could target hepatocytes by recognizing its ligand, asialoglycoprotein receptor (ASGPR), which is an extracellular glycoprotein receptor expressed on the surface of hepatocytes. In addition, the expression of ASGPR is fibrosis stage dependent and could be used for precise treatment in liver fibrosis (Kumar et al., 2021). A work by Zhang et al. (2016) developed a noninvasive method of imaging for SPECT (single-photon emission computed tomography) with an ASGPR targeting tracer-(99m) Tc-p (VLA-co-VNI) to quantify and stage liver fibrosis. Galactose-functionalized polyamidoamine (PAMAM) dendrimer was utilized for hepatocyte targeting by He et al. (2017). Meanwhile, the dendrimer did not show hepatic or renal toxicity, or immunotoxicity, suggesting it may be a safe and efficient hepatocyte-targeting delivery platform. Similarly, pullulan stabilized iron oxide nanoparticles (P-SPIONs) targeting ASGPR were designed for theranostic application of liver diseases (Saraswathy et al., 2021). P-SPIONs showed early diagnosis of liver fibrosis in rodent model. Besides, lipopeptide nanoparticles (LPNP), a kind of apolipoprotein mimicking nanocarriers, have shown promising potential for hepatocyte targeting (Dong et al., 2014). The LPNP effectively and selectively delivered siRNA into hepatocytes via dynamin-dependent micropinocytosis. It is possible that bioinspired design might be a useful strategy for biomaterials as drug delivery systems for liver fibrosis.

**FIGURE 4:**
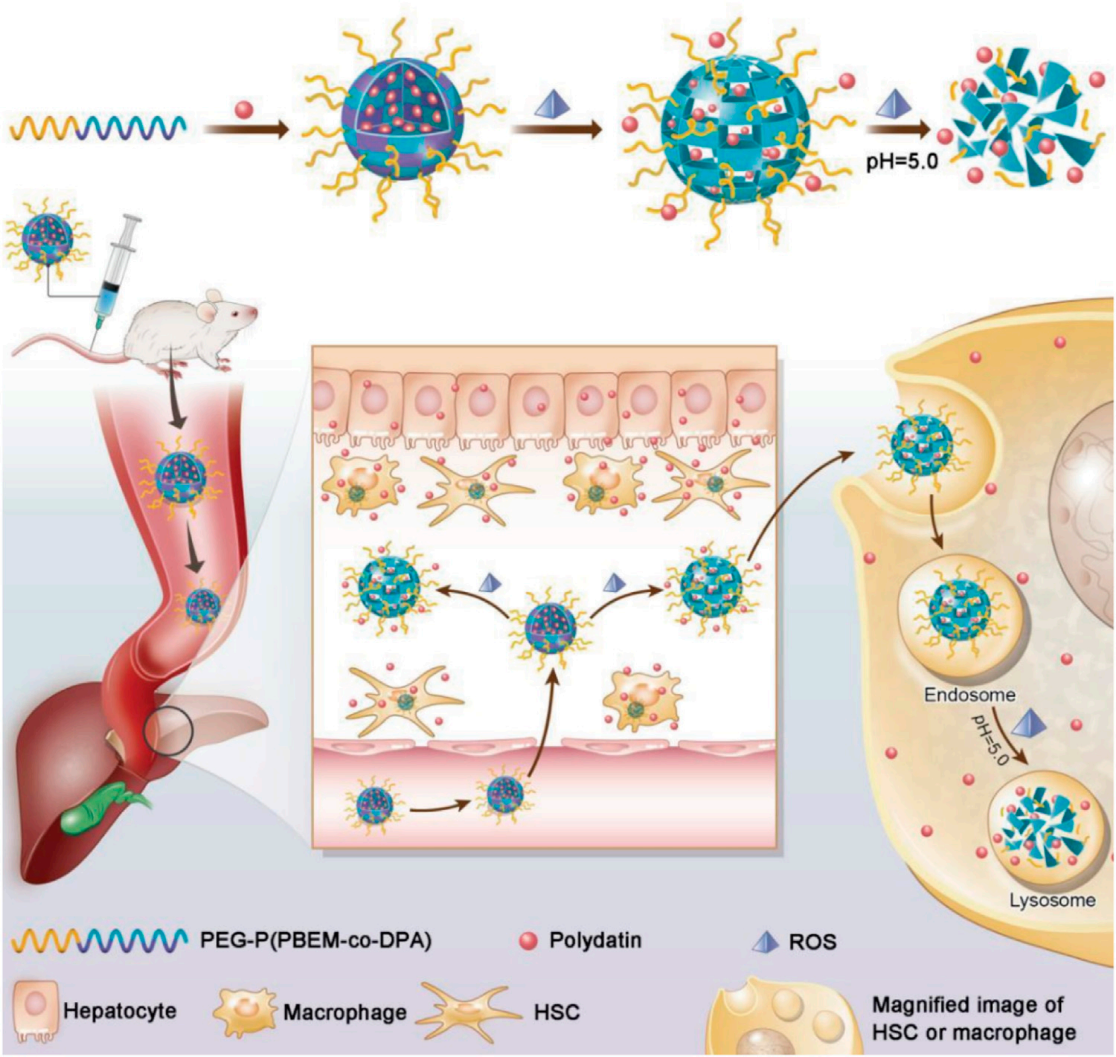
Schematic illustration of PEG-P (PBEM-co-DPA)-Polydatin, a ROS, and pH dual-responsive nanodrug that regulates multiple cell types for the treatment of liver fibrosis (Lin et al., 2020).

In addition, multiple cell-targeting strategies have been applied in fibrosis targeting systems. In view of the over production of ROS in fibrotic livers, a work by Lin et al. (2020) designed PD-MC (polydatin-encapsulated micelle) with ROS and pH dual-sensitivity, realized site-specific drug release in the ROS-rich tissue and the lysosomes (Figure 4). Herein, nano core was synthesized and assembled into the micelle. The PDPA segment was pH-sensitive and let polydatin intracellularly release in the acidic lysosomes (pH 4.5–5.5), while the PPBEM hydrophobic core encapsulating polydatin reacted with ROS to trigger drug release and decrease ROS in liver fibrosis. PD-MC targeted multiple types of hepatic cells, effectively ameliorated liver fibrosis by inhibiting inflammatory response and ROS, prevented hepatocyte apoptosis and averted activation of HSCs and macrophages.

## Nanodrug Delivery System Targeting Liver Sinusoidal Endothelial Cells

As mentioned above, LSECs undergo the loss of endothelial permeability in CLD. Recently a study showed that TiO_2_ NPs could restore sinusoidal permeability by inducing transient leakiness in primary human hepatic sinusoidal endothelial cells (HHSECs), which could improve hepatic recovery and upregulate drug uptake (Tee et al., 2018).

Moreover, in blood, over 90% of hyaluronic acid (HA) is taken and metabolized by LSECs due to the receptors on them (DeLeve and Maretti-Mira, 2017). Therefore, HA modified delivery systems can be used for LSEC targeting in liver fibrosis. Ohya et al. developed biodegradable polyanion-coated polymeric micelles conjugated with HA by polyion complex (PIC) formation, which were taken up only into LSECs (Ohya et al., 2011).

**FIGURE 5:**
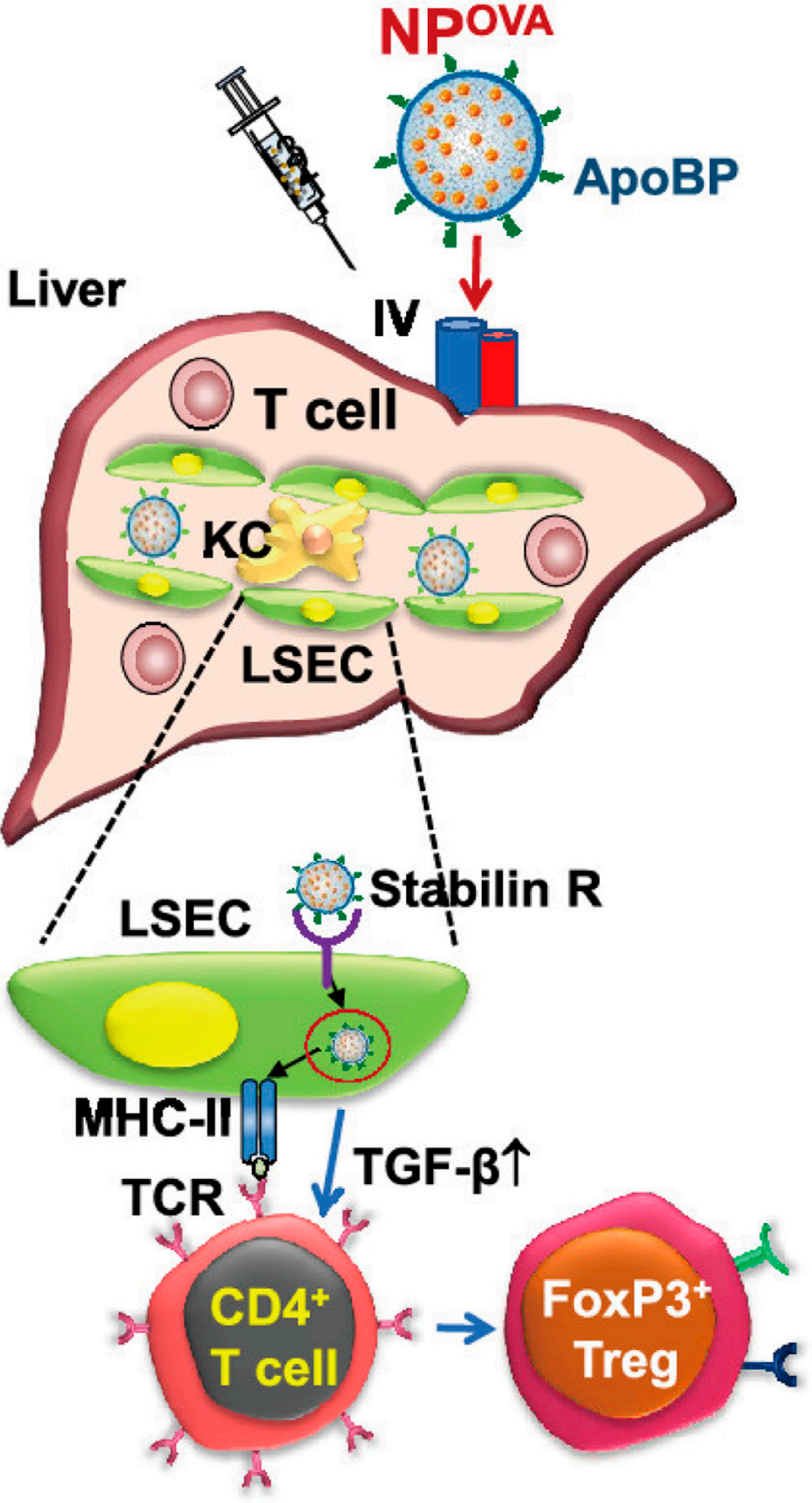
Schematic diagram of the working model for liver-targeting tolerogenic nanoparticles. NPs attached ApoBP ligand delivering the antigens are epitopes to LSECs in the liver through endocytic uptake. Antigen processing and presentation to naive T-cells could generate Foxp3^+^ Tregs, which are recruited to the site of pathology, where they exert immunosuppressive effects (Liu et al., 2021).

It has been reported that rather than hepatocytes, LSECs play the key role in the initial uptake of virus into the liver in a hepatitis B virus model. Fluorescent viral particles and virus protein-coated gold particles exhibited a preferential uptake of the viral substrates into LSECs (Breiner et al., 2001). Thus, utilizing viral pathways of cell targeting in liver might be a candidate for drug delivery to fibrotic liver. Abel et al. prepared mouse CD105-specific lentiviral vectors (mCD105-LV) that can specifically transduce LSECs (Abel et al., 2013). Another work took use of the endocytic uptake by LSECs to deliver antigens to the liver (Liu et al., 2021). The tolerogenic nanoparticles (TNPs) attached ApoBP ligand presented antigens to regulate the differentiation of naive T-cells into Tregs (Figure 5). It seems that LSECs-targeting antigen delivery might be a hopeful candidate in the immune regulation for autoimmune disorders, such as autoimmune hepatitis.

## Conclusion

Liver fibrosis represents a common stage of CLD with a major impact on the human population, and there is an eager need for novel treatments to block and reverse the underlying pathological process. Given a vast number of drugs for liver fibrosis are under investigation, the arsenal of medicines being available is likely to prominently expand in the coming years. Up to now, most anti-fibrotic agents used clinically are neither liver nor fibrosis specific. Nanotechnology provides an opportunity to change this scenario.

However, up till now, the only nanodrug delivery system in the clinical stage for the treatment of liver fibrosis was lipid-based NPs. The targeted lipid nanoparticle delivers HSP47 (heat shock protein 47) siRNA to HSCs in Japanese subjects with moderate to extensive fibrosis (Sakamoto et al., 2018). It was in clinical phase 1b/2 and the results were safe and effective. It cannot be denied that there is still a long way to go before the clinical transformation of nanomedicine.

Firstly, although NPs have exhibited great therapeutic potential for liver fibrosis in preclinical experiments, they also show hepatotoxicity. It has been revealed that exposure to NPs could increase hepatotoxicity (Jia et al., 2017; Li et al., 2018a). The systemically evaluation is need for long-term hepatotoxicity of NPs, particularly when NPs are used in patients with CLD. Besides, it has been reported that NP structures may have strong immunomodulating activity, which can induce both immunostimulation, and immunosuppression (Di Gioacchino et al., 2011). The control of the immunological properties is also necessary for using NPs. Therefore, in preclinical and clinical stages, the safety involved in the use of NPs for liver fibrosis therapy deserve significant attention.

Secondly, many nanodrugs failed clinical development due to their failure in demonstrating a significant improvement in efficacy (Ventola, 2017). Most of the nanodrugs approved have demonstrated reduced toxicity rather than improved efficacy compared to conventional formulations. This may attribute to the difference between experimental models and human, or the lack of assurance of the quality of final products. Moreover, regulation regarding nanopharmaceuticals is still limited (Souto et al., 2020). Cost-benefit considerations should not be neglected as well. Hence, experimental animal models which can more appropriately reflect human pathophysiological processes need to be developed. To ensure the quality, efficacy, and safety of NPs for human use, clinical trials are mandatory (Souto et al., 2020). A reproducible, scalable production method must be developed and validated as well. Last but not least, to avoid investing in developing unlikely approved NPs, comparison with competitor products and a cost-benefit analysis are required.

Thirdly, it must be pointed that therapies based merely on antifibrotic effects cannot stop the driving factors of disease process such as cell stress, inflammation, and apoptosis. In addition, most research focuses on the activation process of HSCs and inflammatory pathways, but neglect the crosstalk between different cell types and different organs. It is also imperative to treat not only the etiology but also the complications of the liver disease, such as hypertension, HE, etc. Maybe no one-medication-fits-all strategy will be successful and the future focus should be on combination therapy. Relative to traditional methods, nanomedicine might be easier to realize one-medication-fits-all strategy for liver fibrosis. Current nanotechnology particularly lipid-based RNA nanomedicines, provide new avenues through acting intracellular. Once establishing a definite mechanism route inside a cell type, lipid-based RNA nanomedicines will largely work independent of binding sites and other factors. Co-delivery of different RNAs will easily achieve the goal of multi-targeting therapy. Besides, microfluidic technology has greatly enriched the fabrication of RNA-based nanomedicines and are hoped to further do so.

In a word, nanotechnology has opened an exciting and promising field of research for the treatment of CLD. It can be envisioned that tailoring nanomedicine delivery systems will allow specific targeting to crucial cell types and achieve combination therapy, with low systemic toxicity. Nevertheless, the clinical translation of nanomedicine for antifibrotic therapies remains elusive and greater advance of our understanding on the potential targets for liver fibrosis is in need for the discovering of promising antifibrotic drug candidates. When overcoming the challenges of nanomedicine such as specific targeting, scale-up and manufacturing, regulatory and safety, it may only be a matter of time until nanomedicine alter clinical practice.
